# Effects of suicide prevention videos developed by and targeting adolescents: a randomized controlled trial

**DOI:** 10.1007/s00787-021-01911-6

**Published:** 2021-11-24

**Authors:** Marlies Braun, Benedikt Till, Jane Pirkis, Thomas Niederkrotenthaler

**Affiliations:** 1 Unit Suicide Research & Mental Health Promotion, Department of Social and Preventive Medicine, Center for Public Health, Medical University of Vienna, Kinderspitalgasse 15, 1090 Vienna, Austria; 2 Centre for Mental Health, Melbourne School of Population and Global Health, The University of Melbourne, Melbourne, Australia

**Keywords:** Suicide prevention, Media, Adolescents, Randomized controlled trial, Papageno effect

## Abstract

Suicide prevention videos featuring young people’s personal narratives of hope and recovery are increasingly used in suicide prevention, but research on their effects is scarce. A double-blind randomized controlled trial was conducted to test the effects of a suicide prevention video featuring an adolescent mastering his suicidal ideation by getting help on 14 to 19-year-olds. *N* = 299 adolescents were randomly allocated to watch the intervention video (*n* = 148) or a control video unrelated to mental health (*n* = 151). Questionnaire data were collected before (*T*_1_) and immediately after exposure (*T*_2_), and 4 weeks later (*T*_3_). Data were analyzed with a repeated-measures ANCOVA. The primary outcome was suicidal ideation, assessed with the Reasons for Living Inventory for Adolescents. Secondary outcomes were help-seeking intentions, attitudes towards suicide, stigmatization of suicidality, and mood. There was an immediate beneficial effect of the intervention on suicidal ideation (*T*_2_ mean change from baseline within intervention group *M*_Change_ = − 0.16 [95% CI − 0.20 to − 0.12], mean difference compared to control group *M*_Diff_ = − 0.09 [95% CI − 0.15 to − 0.03], *η*_p_^2^ = 0.03), which was not maintained at *T*_3_. Participants reported significantly higher help-seeking intentions, which was maintained at 4-week follow-up. They also reported a sustained reduction of favorable attitudes to suicide. Effects on suicidal ideation were mediated by identification with the featured protagonist. Adolescents appear to benefit from suicide prevention narratives featuring personal stories from peers on coping with suicidal ideation and help-seeking.

**Trial registration** DRKS00017405; 24/09/19; retrospectively registered.

## Introduction

Youth suicide is a major public health problem in many countries [1] and ranks consistently among the three most common causes of death among young people [2]. Consequently, considerable attention has been given to how youth suicide might best be combatted. One promising intervention path is media interventions [3]. In recent years, videos featuring personal stories of hope and recovery from suicidal crises have increasingly been used for prevention and education purposes. Several studies suggest that media stories featuring positive narratives of coping might reduce suicidal ideation [4, 5] and increase help-seeking intentions [6], the so-called *Papageno effect* [7]. However, little is known about the impact of these narratives for young people [8, 9]. There are currently only three randomized controlled trials (RCTs) available that investigated media effects of this kind of messaging in young adults [8, 10, 11], but results are inconsistent, and none of these studies have included young adolescents under the age of 18. This is in spite of the fact that this age group is confronted with youth-specific challenges, such as managing identity formation, peer pressure, and developmental changes [12, 13], and the need for tailored prevention strategies has been highlighted in the literature [12, 14]. Media stories explicitly targeting a young audience and providing opportunities for identification might resonate better with this group than other approaches [15]. Video campaigns might be a particularly powerful prevention approach for this age group, as young people commonly use video platforms, such as YouTube, as sources for information [16].

In the present study, we aimed to investigate short- and medium-term effects of a suicide prevention video developed by adolescents for other adolescents who experience suicidal ideation regarding several mental health-related outcomes, including suicidal ideation (primary outcomes), help-seeking intentions, attitudes to suicide, and suicide stigma (secondary outcomes). We hypothesized that the intervention video would have a beneficial impact on these outcomes. Further, in accordance with previous studies, we hypothesized that the effect on suicidal ideation would be stronger the more youth identified with the story [17, 18], and the more vulnerable they were to suicide [4, 11, 18]. We also explored gender differences.

## Methods

### Participants

We conducted a double-blind RCT. Participants were recruited from July 2019 (01/07/19) to October 2020 (12/10/20). Youth aged 14–19 years were invited to participate in a study on health-related awareness videos; more specific details on the aim of the study were not provided and there was no mention of suicide. Invitation flyers were provided at locations in Vienna, Austria, including schools, university events, and online. The announcement stated that participants were required to come to the study center twice, with a 4-week time span between visits, with a compensation of 10€ per visit. Due to the COVID-19 lockdown in spring 2020, participation via an online questionnaire was additionally established to allow those who were already enrolled to complete their 4-week follow-up online.

At the beginning of the trial, inclusion criteria (i.e., age 14–19 years, good German skills, and Austrian residency/permanently living in Austria) were checked, and included individuals completed the Beck Hopelessness Scale [19, 20] to determine individual vulnerability to suicide. Hopelessness has been found to be a robust predictor of suicide risk [21, 22]. For participants who scored above the cut-off score of 33, the researcher further assessed their well-being and immediate suicide risk and informed them about available help services. No participant was excluded in this process. Individual written informed consent was sought from youth and, for minors (under 18-year-olds), additionally from parents.

### Randomization and blinding

Participants were provided with access to a computer workstation. Participants completed the questionnaire on a computer in the online tool SoSci Survey (www.soscisurvey.de). Participants were randomly allocated to view the intervention or control video. They were randomized individually using an automated algorithm in the online tool SoSci Survey. The algorithm applied urn randomization, i.e., a simple 1:1 randomization based on a computerized algorithm (https://www.soscisurvey.de/help/doku.php/en:create:random_urns) [23].

### Materials and procedure

#### Intervention and control video

We tested a suicide prevention video that was produced by students within a school project in 2018/2019, supervised by the lead researcher [24]. In this project, 18 high school students aged 15–19 years produced suicide prevention videos in teams. In total, seven short films were produced. All videos featured young people engaging in various sorts of help-seeking. An international jury of suicide prevention experts selected the best video based on predefined quality criteria, and this video was the intervention video. For further details see Braun et al. [24], https://econtent.hogrefe.com/doi/suppl/10.1027/0227-5910/a000696/suppl_file/0227-5910_a000696_esm2.mp4.

The video had a duration of 4.51 min and featured a 17-year-old boy telling his personal story about his past suicidal crisis and his way of mastering it. The protagonist describes the circumstances that contributed to his suicidal crisis, putting emphasis on how he came through it, noting the relevance of proactive help-seeking, and highlighting different sources of help (i.e., teacher, friends, and professional help). At the end of the video, a list of mental health services for youth was presented.

The control video had a duration of 3.54 min and focused on a personal narrative about a topic unrelated to mental health. It featured the same protagonist talking about nutrition, how he stays fit, and maintains a healthy lifestyle. At the end of the video, a list of references about nutrition and healthy lifestyle was presented. The intervention and the control video were similar in terms of style and production (see Braun et al. [24] for details).

#### Procedure

Before watching the video (*T*_1_), socio-demographics, suicidal ideation, and all secondary outcomes (i.e., help-seeking intentions, attitudes towards suicide, stigma of suicide, and mood) were measured. Immediately after video exposure (*T*_2_), all outcomes were measured again and individuals’ identification with the protagonist was assessed. All participants received a link to their video via e-mail, were encouraged to rewatch the video, and got an e-mail reminder every week. The number of individual viewings was automatically recorded in a database. At 4-week follow-up (*T*_3_), all outcome measures were assessed again, including an item to assess blinding success.

### Primary outcome measure

#### Suicidal ideation

Suicidal ideation was assessed with the Reasons for Living Inventory for Adolescents (RFL-A [25]). It consists of 32 self-report items (e.g., “I am afraid of killing myself”) evaluating a range of adaptive beliefs and reasons for living. A rating scale from 1 (not at all important) to 6 (extremely important) was used. The RFL-A has been identified as a valid and reliable measure of adolescent suicide risk potential [26], and several versions of the RFL have been used in similar media-related studies [4, 10, 18]. Scores were reverse-coded, with higher scores indicating higher suicidal ideation (Cronbach’s *α* = 0.93).

### Secondary outcome measures

#### Help-seeking intentions

The 10-item General Help-seeking Questionnaire (GHSQ [27]) asks respondents to indicate the likelihood of seeking help in the case of suicidal thoughts from a variety of sources. Items were rated on a Likert scale from 1 (extremely unlikely) to 7 (extremely likely) (overall sum core: Cronbach’s *α* = 0.67) and included subscales for private (4 items, Cronbach’s *α* = 0.71) and professional help-seeking intentions (3 items, Cronbach’s *α* = 0.70). Higher scores indicate greater help-seeking intentions.

#### Attitudes towards suicide

Attitudes towards suicide were measured with the Cognitions Concerning Suicide Scale (CCSS [28, 29]). Respondents rated their level of agreement with 20 statements (e.g., “Everyone has the right to commit suicide”) on a Likert scale from 1 (disagree) to 6 (agree). Higher scores indicate more favorable attitudes toward suicide (Cronbach’s *α* = 0.83).

#### Stigma of suicide

The 12-item short version of the Stigma of Suicide Scale (SOSS [30]) was used to assess stigma. Items were rated on a Likert scale from 1 (strongly disagree) to 5 (strongly agree). Scores for the two subscales “Stigma” (8 items, Cronbach’s *α* = 0.87) and “Glorification/Normalization” (4 items, Cronbach’s *α* = 0.73) were calculated. Higher scores indicate greater stigmatizing attitudes toward suicidal individuals.

#### Mood

Current mood was assessed using the”Mood” subscale of the Affective State Scale (ASS [31]), which uses responses to eight adjectives describing respondents’ mood, such as “merry” or “sad”, scored on a scale from 1 (not at all) to 4 (highly). Higher scores indicate better mood (Cronbach’s *α* = 0.87).

### Additional measures

#### Vulnerability

The Beck Hopelessness Scale by Beck and Steer [19], a 10-item measure to assess hopelessness (e.g., “My future seems dark to me”), was used to assess vulnerability at* T*_1_. Based on Krampen [20], a scale rating items from 1 (very false) to 6 (very true) was used. In accordance with previous studies [11], this variable was used to assess vulnerability to suicide. For this purpose, the sample was stratified into low vs. higher vulnerability based on the sample mean (Cronbach’s *α* = 0.77).

#### Identification

Identification was assessed with Cohen’s Identification Scale [32]. Respondents rated their level of agreement with 10 statements (e.g., “While watching the video I could feel the emotions of character X”) on a scale from 1 (completely disagree) to 5 (completely agree). Higher scores indicate greater identification with the protagonist featured in the video (Cronbach’s *α* = 79).

#### Blinding success

To assess blinding success, respondents were asked at *T*_3_ to indicate what group they thought they had been allocated to (“intervention group”, “control group”, or “don’t know”) [4, 33].

### Power analysis

The required sample size was calculated for a repeated-measures analysis of covariance (ANCOVA) with three measurements, a power of 0.80, a two-sided significance level of *p* < 0.05. We had 8 (2 × 2 × 2) statistical groups (i.e., two study groups: intervention versus control; two groups for identification with the protagonist: low [i.e., below the sample mean] versus high [i.e., above the sample mean]; and two groups for suicide baseline vulnerability: low [i.e., below the sample mean] versus high [i.e., above the sample mean]). Assuming a correlation of 0.79 between measuring points, 288 participants were required to detect a moderate intervention effect on suicidal ideation (Cohen’s *f* = 0.21) [5, 11, 18].

### Data analysis

Mean scores were calculated for each outcome variable. Differences in the primary outcome (i.e., suicidal ideation) and in the secondary outcomes between intervention and control group were calculated using a repeated-measures analysis of covariance (ANCOVA). The models were controlled for age, gender, and for baseline scores. Bonferroni-adjusted contrast tests were used to compare individual group differences.

Differences in males and females were explored by adding gender as between-subjects independent variable to the model. To examine the effects of individuals’ vulnerability and identification, the dichotomized variables for suicide baseline vulnerability and identification were added as between-subjects independent variables to the model. Further, a mediation analysis using *PROCESS* [34] was conducted to assess whether identification with the protagonist mediated the effect of the intervention. Model 4 and a bootstrapping method with 5000 bootstrap resamples was used. The bootstrapping method produced 95% bias-corrected confidence intervals of these effects [34].

### Sensitivity analysis

To check whether the findings were different in minors (i.e., participants under the age of 18), a sensitivity analysis was conducted by reanalyzing the data in this subset of participants. Patterns in 14- to 17-year-olds were similar to the presented findings in the full sample (results available on request).

### Ethics statement

All procedures contributing to this work comply with the ethical standards of the relevant national and institutional committees on human experimentation and with the Helsinki Declaration of 1975, as revised in 2008. All procedures involving human participants were approved by the research ethics board at the Medical University of Vienna (study protocol 1033/2015). Individual written consent was sought from all participants and for minors additionally from parents. Trial registration: German Clinical Trial Registry (DRKS00017405; 24 September 2019; retrospectively registered).

## Results

### Study participant characteristics

**Fig. 1:**
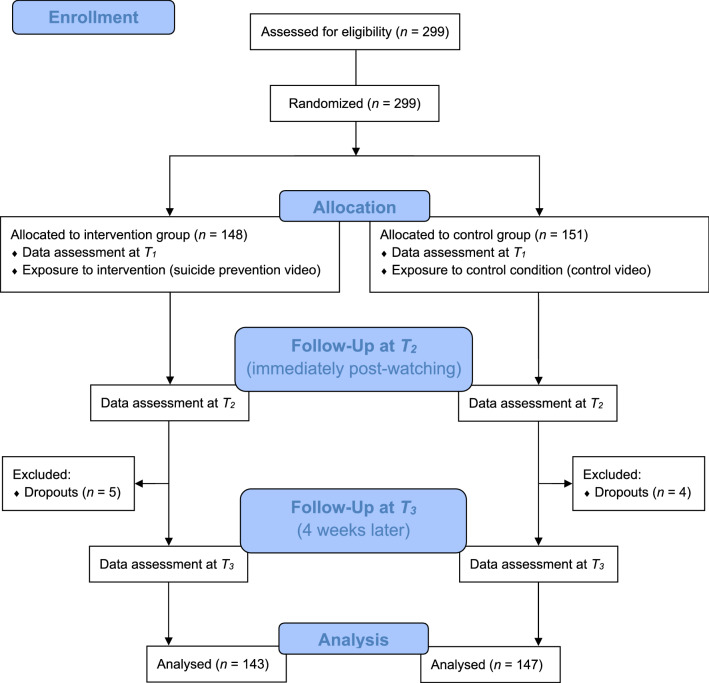
Study flowchart

In total, 299 individuals were randomized (intervention group: *n* = 148 and control group: *n* = 151). *N* = 9 individuals did not provide data at 4-week follow-up, resulting in 290 individuals who completed the questionnaire (96.99%) and were analyzed (see study flowchart: Fig. 1). Out of the 299 participants, *n* = 225 participants were female (75.25%) and *n* = 202 participants (67.56%) had Austrian nationality. The mean age was 17.95 years (SD = 1.19), ranging from 14 up to 19 years, with *n* = 82 (27.42%) individuals being under 18 years old (see Table 1). *N* = 53 (17.73%) participants scored above the cut-off score at the baseline (*T*_1_) screening for vulnerability to suicide. *N* = 39 (13.45%) individuals completed their participation at* T*_3_ online. *N* = 46 (15.38%) participants watched the video more than once. Baseline characteristics of participants were similar between groups as indicated by *χ*^2^ and independent t tests (Table 1). Participants in the intervention group reported more favorable attitudes toward suicide at baseline, identified more with the portrayed character, and were more successful in guessing their group allocation correctly.

**Table 1:** Descriptive statistics for intervention group (*n* = 148) and control group (*n* = 151)

Variable	Group #1: intervention group	Group #2: control group	*χ*^2^/*T*
Gender			
Females *n* (%)	114 (77.03)	111 (73.51)	0.50^a^
Males *n* (%)	34 (22.97)	40 (26.49)	
Age *M* (*SD*)	17.99 (1.21)	17.91 (1.18)	0.57^c^
Nationality			
Austrians *n (%)*	105 (70.95)	97 (64.24)	1.82^b^
Germans *n (%)*	9 (6.08)	9 (5.96)	
Other nationality *n (%)*	34 (22.97)	45 (29.80)	
Occupation			
Student (school) *n* (%)	63 (42.57)	69 (45.70)	2.80^b^
Student (university) *n* (%)	69 (46.62)	58 (38.41)	
Other occupation *n* (%)	16 (10.81)	24 (15.89)	
Suicide attempt in last year *n* (%)	12 (8.11)	8 (5.30)	0.95^a^
Treatment of mental illness at present *n* (%)	4 (2.70)	7 (4.64)	0.79^a^
Recruitment method			
Recruitment via website *n* (%)	60 (40.54)	72 (47.68)	1.77^b^
Recruitment via recommendation *n* (%)	26 (17.57)	26 (17.22)	
Recruitment via other sources *n* (%)	62 (41.89)	53 (35.10)	
Baseline hopelessness (BHS) *M* (SD)	2.57 (0.69)	2.59 (0.75)	− 0.21^c^
Baseline suicidal ideation (RFL-A total) *M* (SD)	2.34 (0.79)	2.35 (0.75)	− 0.11^c^
Baseline *Family Alliance* (RFL-A subscale) *M* (SD)	2.18 (1.22)	2.19 (1.15)	− 0.05^c^
Baseline *Suicide Related Concerns* (RFL-A subscale) *M* (SD)	2.89 (1.38)	2.97 (1.45)	− 0.48^c^
Baseline *Peer Acceptance and Support* (RFL-A subscale) *M* (SD)	2.18 (0.98)	2.07 (0.97)	0.93^c^
Baseline help-seeking (GHSQ total) *M* (SD)	3.70 (1.08)	3.83 (1.04)	− 1.09^c^
Baseline help-seeking private (GHSQ subscale) *M* (SD)	4.03 (1.33)	4.18 (1.25)	− 1.00^c^
Baseline help-seeking professional (GHSQ subscale) *M* (SD)	3.62 (1.35)	3.63 (1.36)	− 0.08^c^
Baseline attitudes toward suicide (CCSS) *M* (SD)	2.63 (0.76)	2.45 (0.75)	2.13^ cd^
Baseline stigma towards suicide (SOSS subscale) *M* (SD)	2.17 (0.89)	2.16 (0.88)	0.08^c^
Baseline normalization/glorification of suicide (SOSS subscale) *M* (SD)	2.04 (0.86)	1.99 (0.81)	0.50^c^
Baseline mood (ASS) *M* (SD)	2.99 (0.59)	3.03 (0.60)	− 0.48^c^
Identification with protagonist featured in video *M* (*SD*)	3.56 (0.74)	3.38 (0.67)	2.30^cd^
Multiple video viewing *n* (%)	24 (16.22)	22 (14.57)	0.16^a^
Completed questionnaire *n* (%)	143 (96.62)	147 (97.35)	0.14^a^
Manipulation check correct *n* (%)	50 (34.97)	36 (24.49)	7.37^bd^

Frequencies (*n*), percentages (%), means (*M*), and standard deviations (SD) provided for each group, as well as *χ*^2^ values from *χ*^2^ tests and *t* values from independent t tests testing group differences

^a^*χ*^2^ test result. *df* = 1

^b^*χ*^2^ test result. *df* = 2

^c^Independent *t* test result

^d^*p* < 0.05

#### Differences between dropouts and study completers

We used independent *t* tests and Fisher’s exact tests to analyze whether participants who dropped out (*n* = 9) were different from study completers (*n* = 200). Austrian nationality (*χ*^2^(2) = 6.16, *p* = 0.04), completed high school degree (*χ*^2^(2) = 9.51, *p* < 0.01), and high school students (*χ*^2^(2) = 10.49, *p* < 0.01) were underrepresented among dropouts with no further differences in socio-demographics or baseline (*T*_1_) measurements of outcome variables.

### Blinding

86 participants (29.66%) guessed their group allocation correctly, 87 (30.00%) were incorrect, and 117 (40.34%), responded with “don’t know”. More than half of the participants in each group were either uncertain or incorrect about their group assignment, with the majority of participants responding with “don’t know” in both, intervention and control group respectively. There were no differences between the two study arms in correctly guessing the group allocation (*χ*^2^(1) = 3.81, *p* > 0.05).

### Suicidal ideation

Table 2 compares outcomes in both study arms. Suicidal ideation scores at *T*_2_ were significantly lower in the intervention group (*T*_2_ mean change from baseline within intervention group *M*_Change_ = − 0.16 [95% CI − 0.20 to − 0.12], mean difference compared with control group M_Diff_ = − 0.09 [95% CI − 0.15 to − 0.03], η_p_^2^ = 0.03). This small effect was not maintained at 4-week follow-up (*T*_3_ *M*_Change_ = − 0.09 [95% CI − 0.16 to − 0.03], M_Diff_ = − 0.09 [95% CI − 0.19 to 0.00]). Findings for the suicidal ideation subscale *Peer Acceptance and Support* revealed a similar pattern, with significantly lower scores in the intervention group (*T*_2_ *M*_Change_ = − 0.25 [95% CI − 0.33 to − 0.17], *M*_Diff_ = − 0.20 [95% CI − 0.30 to − 0.10], *η*_p_^2^ = 0.05), which was maintained 4 weeks later (*T*_3_ *M*_Change_ = − 0.08 [95% CI − 0.18 to 0.01], *M*_Diff_ = − 0.18 [95% CI − 0.32 to − 0.04], *η*_p_^2^ = 0.02).

**Table 2:** Changes in suicidal ideation and secondary outcomes in the intervention group (suicide prevention video) and control group (control video) from baseline to after video exposure (*T*_2_) and 4 weeks later (*T*_3_) among all participants

Outcome	After video exposure (*T*_2_*, n* = 299)^a^						4 weeks later (*T*_3_, *n* = 290)^b^					
	Mean (SD)	Mean change (95% CI)^c^ from baseline	Mean difference (95% CI)^d^	*t* test	*p*	*η*_p_^2^	Mean (SD)	Mean change (95% CI)^c^ from baseline	Mean difference (95% CI)^d^	*t* test	*p*	*η*_p_^2^
Suicidal ideation (RFL-A total)												
Control group	2.29 (0.78)	− **0.07 (**− **0.11, **− 0.03)***					2.35 (0.78)	0.00 (− 0.08, 0.07)				
Intervention group	2.18 (0.85)	− **0.16 (**− **0.20, **− 0.12)***	− 0.09 (− 0.15, − 0.03)	− 3.08	**< 0.01**	0.03	2.26 (0.84)	− **0.09 (**− **0.16, **− 0.03)**	− 0.09 (− 0.19, 0.00)	− 1.87	0.06	0.01
Peer acceptance and support (RFL-A subscale)												
Control group	2.03 (1.06)	− 0.04 (− 0.10, 0.02)					2.20 (1.01)	0.11 (0.00, 0.22)*				
Intervention group	1.92 (0.97)	− **0.25 (**− **0.33, **− 0.17)***	− 0.20 (− 0.30, − 0.10)	− 4.02	**< 0.001**	0.05	2.10 (0.99)	− 0.08 (− 0.18, 0.01)	− 0.18 (− 0.32, − 0.04)	− 2.48	**0.01**	0.02
Future optimism (RFL-A subscale)												
Control group	2.15 (0.98)	− **0.18 (**− **0.24, **− 0.11)***					2.25 (1.02)	− 0.06 (− 0.18, 0.05)				
Intervention group	1.93 (0.85)	− **0.19 (**− **0.26, **− 0.12)***	− 0.04 (− 0.13, 0.05)	− 0.81	0.42	< 0.01	2.06 (0.97)	− 0.08 (− 0.19, 0.03)	− 0.05 (− 0.20, 0.10)	− 0.64	0.53	< 0.01
Help-seeking (GHSQ)												
Control group	3.85 (1.09)	0.01 (− 0.04, 0.07)					3.95 (1.05)	0.11 (− 0.02, 0.25)				
Intervention group	3.92 (1.13)	0.22 (0.13, 0.31)***	0.20 (0.10, 0.30)	3.87	**< 0.001**	0.05	3.99 (1.12)	0.30 (0.15, 0.45)***	0.15 (− 0.04, 0.33)	1.53	0.13	0.01
Help-seeking private (GHSQ subscale)												
Control group	4.25 (1.32)	0.07 (− 0.01, 0.14)					4.37 (1.27)	0.19 (0.03, 0.36)*				
Intervention group	4.30 (1.41)	0.27 (0.15, 0.39)***	0.20 (0.06, 0.34)	2.85	**< 0.01**	0.03	4.37 (1.28)	0.34 (0.16, 0.52)***	0.11 (− 0.11, 0.33)	0.99	0.32	< 0.01
Help-seeking professional (GHSQ subscale)												
Control group	3.60 (1.37)	− 0.03 (− 0.13, 0.06)					3.67 (1.36)	0.03 (− 0.14, 0.20)				
Intervention group	3.79 (1.35)	0.17 (0.05, 0.29)**	0.20 (0.05, 0.34)	2.65	**0.01**	0.02	3.89 (1.43)	0.28 (0.09, 0.48)**	0.25 (0.00, 0.49)	2.00	**0.05**	0.01
Attitudes towards suicide (CCSS)												
Control group	2.48 (0.77)	0.03 (− 0.03, 0.09)					2.58 (0.74)	0.12 (0.03, 0.21)**				
Intervention group	2.56 (0.77)	− **0.07 (**− **0.13, **− 0.02)*	− 0.09 (− 0.17, 0.00)	− 2.06	**0.04**	0.01	2.59 (0.76)	− 0.04 (− 0.11, 0.04)	− 0.12 (− 0.23, − 0.01)	− 2.07	**0.04**	0.01
Stigma of suicide (SOSS subscale)												
Control group	2.10 (0.89)	− **0.07 (**− **0.12, **− 0.01)*					2.11 (0.89)	− 0.02 (− 0.11, 0.07)				
Intervention group	2.07 (0.87)	− **0.10 (**− **0.17, **− 0.03)**	− 0.03 (− 0.12, 0.05)	− 0.79	0.43	< 0.01	2.17 (0.91)	0.01 (− 0.09, 0.11)	0.04 (− 0.08, 0.17)	0.69	0.49	< 0.01
Glorification/normalization of suicide (SOSS subscale)												
Control group	1.97 (0.84)	− 0.02 (− 0.08, 0.05)					2.19 (0.91)	0.19 (0.07, 0.32)**				
Intervention group	1.97 (0.88)	− 0.07 (− 0.16, 0.01)	− 0.05 (− 0.15, 0.06)	− 0.85	0.39	< 0.01	2.07 (0.93)	0.05 (− 0.08, 0.18)	− 0.13 (− 0.30, 0.03)	− 1.59	0.11	0.01
Mood (ASS)												
Control group	3.07 (0.61)	0.04 (− 0.01, 0.10)					3.08 (0.60)	0.06 (− 0.03, 0.15)				
Intervention group	2.94 (0.64)	− 0.06 (− 0.12, 0.01)	− 0.10 (− 0.19, − 0.02)	− 2.38	**0.02**	0.02	3.14 (0.51)	0.15 (0.05, 0.24)**	0.07 (− 0.04, 0.18)	1.33	0.19	0.01

*p < 0.05. **p < 0.01. ***p < 0.001 (two-tailed). Significant *p* values are in bold

^a^Control group *n* = 151; Intervention group *n* = 148

^b^Control group *n* = 147; Intervention group *n* = 143

^c^Comparison of means after exposure with the baseline mean with Bonferroni-corrected contrast tests

^d^Comparison of means for the intervention group with the control group with Bonferroni-corrected contrast tests

### Secondary outcomes

Participants in the intervention group reported significantly higher likelihood of seeking help in general at *T*_2_ (*T*_2_ *M*_Change_ = 0.22 [95% CI 0.13–0.31], *M*_Diff_ = 0.20 [95% CI 0.10–0.30], *η*_p_^2^ = 0.05). This small effect was not maintained at 4-week follow-up (*T*_3_ *M*_Change_ = 0.30 [95% CI 0.15–0.45], *M*_Diff_ = 0.15 [95% CI − 0.04 to 0.33). There was an immediate significant small effect on help-seeking intentions from private sources (*T*_2_ *M*_Change_ = 0.27 [95% CI 0.15–0.39], *M*_Diff_ = 0.20 [95% CI 0.06–0.34], *η*_p_^2^ = 0.03), which not maintained at 4-week follow-up (*T*_3_ *M*_Change_ = 0.34 [95% CI 0.16–0.52], *M*_Diff_ = 0.11 [95% CI − 0.11 to 0.33). Participants in the intervention group also had higher help-seeking intentions from professional sources (*T*_2_ *M*_Change_ = 0.17 [95% CI 0.05–0.29], *M*_Diff_ = 0.20 [95% CI 0.05–0.34], *η*_p_^2^ = 0.02), which was maintained at 4-week follow-up (*T*_3_ *M*_Change_ = 0.28 [95% CI 0.09–0.48], *M*_Diff_ = 0.25 [95% CI 0.00–0.49], *η*_p_^2^ = 0.01).

Participants in the intervention group showed a small decrease in favorable attitudes towards suicide (*T*_2_ *M*_Change_ = − 0.07 [95% CI − 0.13 to − 0.02], *M*_Diff_ = − 0.09 [95% CI − 0.17 to 0.00], *η*_p_^2^ = 0.01). This effect was maintained at 4-week follow-up (*T*_3_ *M*_Change_ = − 0.04 [95% CI − 0.11 to 0.04 *M*_Diff_ = − 0.12 [95% CI − 0.23 to − 0.01], *η*_p_^2^ = 0.01). There was a short-term deterioration in mood in the intervention group (*T*_2_ *M*_Change_ = − 0.06 [95% CI − 0.12 to 0.01], *M*_Diff_ = − 0.10 [95% CI − 0.19 to − 0.02], *η*_p_^2^ = 0.02), which was not maintained at 4-week follow-up. There was no significant group effect on the stigmatization of suicide as well as glorification/normalization of suicide.

### Effects of vulnerability

The analysis revealed no significant group × vulnerability interaction with regard to suicidal ideation.

### Effects of identification

There was no significant group × identification interaction with regard to suicidal ideation. The mediation analysis revealed that the relationship between group allocation and suicidal ideation at *T*_2_ was mediated by identification (indirect effect IE = 0.03 [95% CI 0.001–0.08]). Allocation to the intervention group was associated with higher identification (*b* = − 0.19, s.e. = 0.08, *t*(297) = − 2.30, *p* = 0.02), and identification was negatively associated with suicidal ideation (*b* = − 0.18, s.e. = 0.07, *t*(297) = − 2.68, *p* < 0.01).

### Effects of gender

We found no effect of gender with regard to suicidal ideation. The analysis revealed a significant group × gender interaction for intentions to help-seeking in general, *F*(1,293) = 5.21, *p* < 0.05 (see Table 3). Girls/women in the intervention group had higher help-seeking intentions (*T*_2_ *M*_Change_ = 0.26 [95% CI 0.15−0.36], *M*_Diff_ = 0.27 [95% CI 0.15−0.39 ]). A similar pattern was present for help-seeking intentions from private sources (*F*(1,293) = 9.43, *p* < 0.01), but not for help-seeking intentions from professional sources, which did not show any gender differences.

**Table 3:** Changes in outcomes with a significant group × gender interaction in the intervention group (suicide prevention video) and control group (control video) from baseline to after video exposure (*T*_2_) among boys/men and girls/women

Outcome, time after exposure	Mean (SD) after exposure	Mean change (95% CI)^c^ from baseline	Mean difference (95% CI)^d^	*t* test	*p*	*η*_p_^2^	Group × Gender *F* *p*^*e*^
Help-seeking (GHSQ), *T*_2_							
Male^a^							5.21 **0.02**
Control group	4.28 (0.85)	0.08 (− 0.01, 0.18)	0.00 (− 0.18, 0.19)	0.05	0.96	< 0.01	
Intervention group	3.76 (1.25)	0.10 (− 0.06, 0.26)					
Female^b^							
Control group	3.69 (1.13)	− 0.01 (− 0.08, 0.05)	0.27 (0.15, 0.39)	4.35	**< 0.001**	0.08	
Intervention group	3.97 (1.09)	0.26 (0.15, 0.36)***					
Help-seeking private (GHSQ subscale), *T*_2_							
Male^a^							9.43 **< 0.01**
Control group	5.05 (0.90)	0.28 (0.14, 0.42)***	− 0.22 (− 0.48, 0.04)	− 1.69	0.09	0.04	
Intervention group	4.02 (1.56)	0.16 (− 0.05, 0.38)					
Female^b^							
Control group	3.96 (1.33)	− 0.01 (− 0.09, 0.08)	0.32 (0.16, 0.48)	3.94	**< 0.001**	0.07	
Intervention group	4.38 (1.35)	0.30 (0.16, 0.44)***					

*p < 0.05. **p < 0.01. ***p < 0.001 (two-tailed). Significant *p* values are in bold

^a^Control group *n* = 40; Intervention group *n* = 34

^b^Control group *n* = 111; Intervention group *n* = 114

^c^Comparison of means after exposure with the baseline mean with Bonferroni-corrected contrast tests

^d^Comparison of means for the intervention group with the control group with Bonferroni-corrected contrast tests

^e^ANCOVA results, *df* = 1

## Discussion

This study is the first to assess the effects of a short suicide prevention video developed by and targeting adolescents in a randomized controlled trial. Young people benefitted from the brief intervention that included watching a short film featuring an adolescent with past suicidal ideation, describing his story of getting help and recovering. Adolescents reported significantly lower suicidal ideation after watching the intervention video. The video was also effective in increasing help-seeking intentions, which has been highlighted as an essential target domain for suicide prevention [3]. We found that the video increased help-seeking intentions, including from private and professional sources, and the effects were maintained until 4-week follow-up. Immediate effects on help-seeking intentions were present in girls/women but not in boys/men, which is consistent with previous studies, suggesting that males show lower help-seeking [6, 35]. There were, however, no gender differences for the crucial aspect of help-seeking from professional sources, indicating that also boys/men benefitted to some extent in terms of help-seeking intentions. Furthermore, favorable attitudes towards suicide that have previously been associated with higher suicide risk [29], decreased significantly after watching the suicide prevention video.

The findings build on and extend findings from previous studies which suggest a reduction of suicidal ideation after exposure to media stories featuring individuals telling their stories of mastering their crises [4, 5, 11, 18]. In this study, individuals with varying levels of vulnerability to suicide benefitted from the prevention videos. Compared to previous research in the area, we identified a more than seven times higher proportion of individuals with vulnerability to suicide (*N* = 53; 17.73%) using the same measure and cut-off score for vulnerability [11]. This is important because some previous studies in the field have found positive effects on suicidal ideation particularly in individuals with some degree of vulnerability to suicide [4, 10, 11, 18]. Although this is encouraging regarding positive effects in young people who are at risk, a current limitation of the available research including the present study is that it focused on individuals from the general population and all studies, except the present one, included adults [4, 10, 11, 18]. This means that the findings cannot be generalized to young individuals with clinical suicidality. Future studies should aim to assess effects of stories of coping and recovery in young people with some degree of vulnerability based on clinical assessment. Effects in these groups might deviate from the present findings, and specific circumstances and diagnoses might be relevant to any effects.

Unlike previous studies that focused on harmful media impacts of stories of suicide [18], the reduction in suicidal ideation following exposure to the suicide prevention video was mediated by identification. In general, identification with the narrative was high in the intervention group and higher compared to a previous study using the same measure for identification [15]—this might be because the videos were specifically tailored to adolescents and featured a young protagonist. The findings highlight the necessity of producing narratives that the audience can easily identify with to reduce suicidal ideation.

Although there are studies on personal media narratives of hope, which have found a media effect on either suicidal ideation [4, 5] or help-seeking [6], this is the first study that detected preventive effects on both of these crucial outcomes. This finding indicates that it is indeed possible to positively influence both outcomes with the same narrative. The present narrative emphasized both, the individual mastery of the suicidal crisis and the proactive help-seeking and success of help-seeking. At one point, the male protagonist encourages the audience to do the same by saying: “It is important to seek help […]. To get rid of those [suicidal] thoughts, you have to talk to someone. […] Now I know that every human life matters. Mine too. […] It takes a lot of time and effort. However, it pays off”. This clearly links his personal mastery of suicidal ideation to his help-seeking efforts.

### Strengths and limitations

Strengths of this study include the RCT design with a sufficient number of participants and very low dropouts, and blinding was successful. Furthermore, the study was conducted on-site rather than online, increasing its validity [36], and differences between the intervention and the control video were minimal regarding stylistic means. Finally, the assessment included a 4-week follow-up to test for the sustainability of the effects, rather than only immediate effects like in previous studies [5, 10, 18].

The study had some limitations. First, the sample size was only appropriate to detect medium-sized effects. Only a small number of participants watched the video multiple times, so we were not able to calculate effects of repeated exposure. Female adolescents were overrepresented in the study sample. Further, participants were exposed to one intervention video only, and materials should be tailored specifically to various groups, e.g., girls [37]. However, gender patterns suggested that girls/women did not feel less addressed by the male protagonist, but scored better on help-seeking intentions than boys/men. Outcome measures did not capture actual behavior (i.e., suicidal behavior or help-seeking behaviors), which would require larger sample sizes and longer follow-up. Finally, the present sample included a number of youth with some degree of vulnerability to suicide, but it remains unknown if the effects generalize to clinical samples at risk of suicide.

## Conclusion

Adolescents appear to benefit from personal video narratives featuring adolescents who master their suicidal crises and emphasize help-seeking, consistent with the *Papageno effect* [7]. Suicide prevention videos developed by and for adolescents appear to be safe and effective in reducing some risk factors for suicide. These findings should serve as a bold encouragement for public health and school authorities to implement similar projects that put young people in charge of the development of media interventions for suicide prevention.

## Data Availability

The data from this study are available from the corresponding author upon reasonable request.
